# Diffuse Large B Cell Lymphoma Arising in Patients with Preexisting Hodgkin Lymphoma

**DOI:** 10.3390/curroncol29090480

**Published:** 2022-08-25

**Authors:** Emilio Bellitti, Pierluigi Masciopinto, Pellegrino Musto, Elena Arcuti, Luca Mastracci, Giuseppina Opinto, Sabino Ciavarella, Attilio Guarini, Gerardo Cazzato, Giorgina Specchia, Eugenio Maiorano, Francesco Gaudio, Giuseppe Ingravallo

**Affiliations:** 1 Section of Pathology, Department of Emergency and Organ Transplantation (DETO), University of Bari Aldo Moro, 70124 Bari, Italy; 2 Hematology Section, Department of Emergency and Transplantation, University of Bari Medical School, 70124 Bari, Italy; 3 Department of Surgical Sciences and Integrated Diagnostics, University of Genoa, 16126 Genoa, Italy; 4 Haematology and Cell Therapy Unit, IRCCS-Istituto Tumori ‘Giovanni Paolo II’, 70124 Bari, Italy

**Keywords:** classical Hodgkin’s lymphoma, diffuse large B cell lymphoma, cell of origin

## Abstract

The metachronic onset of diffuse large B-cell lymphoma (DLBCL) after classic Hodgkin lymphoma (cHL) is a rare event affecting patients’ outcomes. However, although several studies have investigated the prognostic role of this event, little is known about a hypothetical common origin of the two different neoplastic cells. Aims: To investigate a possible relationship between DLBCL and cHL, in this retrospective study of 269 patients with newly diagnosed cHL treated at Bari University Hospital (Italy) between 2007 and 2020, we analyzed data from 4 patients (3 male and 1 female) with cHL who subsequently developed DLBCL. Methods: Gene expression profile analysis, assessed by NanoString Lymphoma Subtype Assay, was performed to identify the cell of origin in the DLBCL cases, in addition to Hans’s algorithm. Results: Using Hans’s algorithm, all DLBCL cases showed a germinal center-B-Cell subtype. The gene expression profile evaluated by the NanoString Lymphoma Subtype Assay revealed two cases of the GCB molecular subtype, while the others were unclassified. After first-line chemotherapy, 1 patient achieved complete remission, 3 were non-responders (2 died of lymphoma within 6 months, whereas the other achieved complete remission after autologous and allogeneic stem cell transplantation and is still alive). Conclusions: The origin of the second neoplastic cell in patients with DLBCL with a previous history of cHL remains controversial, although the different immunophenotypic characteristics suggest that it may mainly arise *de novo* in a subject with a possible individual predisposition to develop lymphoid neoplasms.

## 1. Introduction

Lymphomas are a variegated group of lymphoproliferative diseases featuring differences in cellular origin, biological development, clinical signs and symptoms and outcome. In fact, they derive from lymphocytes at various stages of development, and the characteristics of the specific subtype of lymphoma mirror those of the cell from which they originated [1]. However, in some cases, a synchronic or metachronic onset of two different lymphomas in the same patient is possible.

Numerous studies have shown that most B-cell lymphomas arise from the germinal center (GC) or post-GC B-cells. This could be due to various mutagenic factors following the vigorous proliferation of GC cells and physiological mutagenic processes (somatic hypermutation and immunoglobulin class change) [1,2,3,4,5].

Progression to diffuse large B cell lymphoma (DLBCL) is a rare event in classical Hodgkin’s lymphoma (cHL), whereas it is more frequent in nodular lymphocyte-predominant Hodgkin lymphoma, although still rare. Even if cHL and DLBCL are considered distinct entities, they could hypothetically represent a continuous spectrum of B cell malignant transformation [6,7,8,9]. Furthermore, the treatment of lymphoid neoplasms induces a state of immunosuppression that could be an important risk factor for the onset of further lymphoid neoplasms [9].

In this study we analyze the possibility of a clonal evolution from cHL to DLBCL, in terms of cellular origin, applying gene expression profile analysis and immunohistochemistry.

## 2. Patients and Methods

This retrospective study was focused on 269 patients with newly diagnosed classic Hodgkin lymphomas treated at Bari University Hospital (Italy) between 2007 and 2020, with the aim of identifying patients who developed DLBCL and verifying a possible common origin. All patients were clinically staged according to the Ann-Arbor system, recording the medical history, complete physical examination, blood counts, biochemical profile, chest X-ray films, PET/CT total body, computed tomography of the chest, abdomen and pelvis, unilateral bone marrow biopsy. Median age at diagnosis was 35 years (range 15–83), 90 pts (33%) had advanced stage (III–IV), 110 (41%) bulky disease, 123 (46%) presented B symptoms, and 71 (26%) extra-nodal disease.

All patients were treated with chemotherapy alone (ABVD) or chemotherapy plus radiotherapy according to guidelines [10].

All cases with a subsequent diagnosis of DLBCL were retrospectively reviewed according to the diagnostic criteria of the WHO 2018 classification of hematopoietic and lymphoid tissue tumors. 

Representative tissue sections, 3 μm thick, were obtained; hematoxylin and eosin staining was performed on one section, while the others were subjected to IHC and in situ hybridization of Epstein-Barr virus (EBV) encoded small RNA (EBER). In addition to the routine pathological examination, PD-L1 was assayed as a critical marker to differentiate Primary mediastinal large B-cell lymphoma from other entities. CD3 and CD5 expression, as well as that of PD-L1, was evaluated to identify some characteristics of the tumor microenvironment (TME).

Cell-of-Origin (COO) was analyzed according to the Hans algorithm and with gene expression profiles. Recently, Scott et al. described a robust model for COO assignment called Lymph2Cx [11]. With nCounter platform of NanoString Technologies we applied Lymph2Cx on formalin-fixed, paraffin embedded RNA. The results obtained are reported as one of two molecular subtypes, Activeted-B-Cell (ABC), or Germinal Center-B-Cell (GC), moroever, the Lymph2Cx assay recognized a group of unclassified cases, where confident assignment cannot be made to either ABC or GCB subtype. With the NanoString Lymphoma Subtype (LST) Assay we evaluated the expression profile of 20 genes within the LST signature, 15 target genes and 5 reference genes. The test was performed on formalin-fixed, paraffin-embedded RNA derived from a 5 μm thick section and the data were analyzed with NanoString Dx nCounter. The results obtained are reported as one of two molecular subtypes, Activated-B-Cell (ABC), or Germinal Center-B-Cell (GCB) or as Unclassified.

Clinical characteristics, including age, gender, clinical stage at presentation, B symptoms and treatments at first diagnosis and at the time of transformation, were recorded and analyzed. First-line therapies included ABVD (doxorubicin, bleomycin, vinblastine, and dacarbazine) after cHL had been diagnosed, and R-CHOP (rituximab cyclophosphamide, doxorubicin, vincristine, and prednisone) when DLBCL developed. The IGEV regimen (Ifosfamide, gemcitabine, and vinorelbine) followed by autologous stem cell transplantation (ASCT) was given as second-line therapy in one patient with relapsed cHL. 

### Follow-Up

After treatment, clinical history, physical examination, and laboratory analysis including full blood cell count, erythrocyte sedimentation rate testing and blood chemistry were repeated at 3 and 6 months, then every 6 months until the 4th year, and once a year thereafter. CT scans and previously pathological radiographic tests were carried out once to confirm the remission status. Subsequently, patients underwent clinical follow-up. 

Cancer screenings were conducted regularly in view of the persistently increased risk of post-treatment development of hematological and solid second malignancies. 

## 3. Results

Over the study period, 269 patients were diagnosed with cHL; in 4 (3 males and 1 female) patients (1.5%), DLBCL developed after a median time of 53 months. Table 1 summarizes the main clinical and laboratory findings in these patients. 

**Figure 1:**
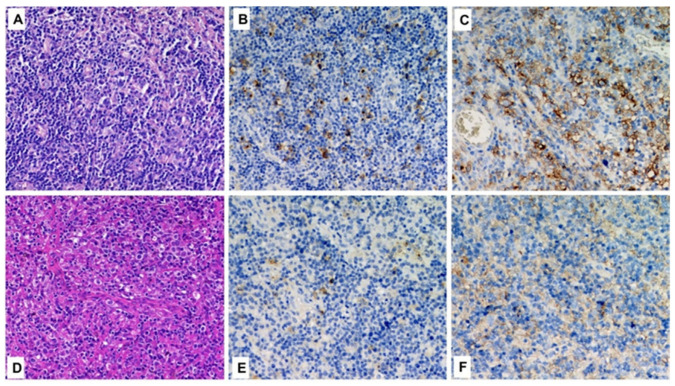
Histological comparison between cHL (**A**–**C**) and DLBCL (**D**–**F**) in the same patient (case n.1). In each row, from left to right, hematoxylin-eosin, CD30 and PD-L1 (original magnification 200×).

**Figure 2:**
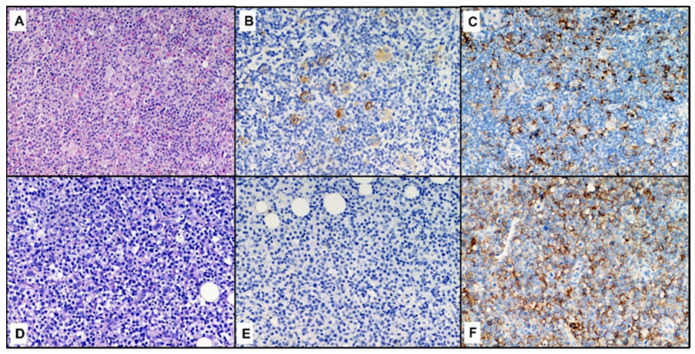
Histological comparison between cHL (**A**–**C**) and DLBCL (**D**–**F**) in the same patient (case n.3). In each row, from left to right, hematoxylin-eosin, CD30 and PD-L1 (original magnification 200×).

**Figure 3:**
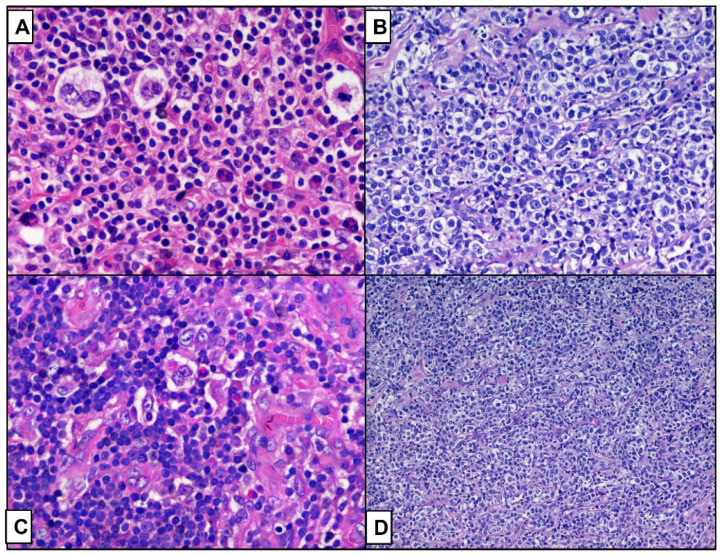
Histological comparison between cHL ((**A**–**C**), hematoxylin-eosin, original magnification 200×) and DLBCL ((**B**–**D**) hematoxylin-eosin, original magnification 200× and 100×), in the patient respectively 2 (**A**,**B**) and 4 (**C**,**D**).

All 4 patients were cHL free at the time of DLBCL diagnosis. Patients 1, 2 and 4 performed, as 1st line for cHL, 6 cycles of ABVD obtaining complete remission. At a median time of 33 months, these showed suspected signs of disease recurrence, but DLBCL was diagnosed on histological examination. Patient number 3, with stage 2A, was treated with 4 cycles of ABVD followed by “involved field” radiotherapy, and subsequently had histologically documented recurrence of cHL; he underwent salvage therapy according to IGEV and ASCT schemes. Lactic dehydrogenase at diagnosis of cHL was normal in all 4 patients. The mean age at presentation with cHL was 28 years (range 18–76 years), and the mean age at which DLBCL was diagnosed was 35 years (range 21–79). Median time from diagnosis of cHL to DLBCL was 32 months (range 24–105). Morphological review of histological slides, as well as immunohistochemical evaluation, confirmed the previous diagnosis (Figure 1, Figure 2 and Figure 3).

All cHL cases tested positive for PD-L1 against a non-neoplastic inflammatory background, while only two patients with DLBCL revealed positivity in a high percentage of neoplastic cells (patients 3 and 4). None of the DLBCLs showed EBV infection detected on EBER. Using Hans’s algorithm, all DLBCL cases showed a non-GBC subtype. No patients expressed CD10, while BCL6 was expressed in three cases (n 1, 2, 3) and MUM1 was expressed in all cases. The gene expression profile evaluated by the NanoString lymphoma subtype tesst showed an origin from GCP in two patients (n 1 and 2), while in cases 3 and 4, it was indeterminate (patients with PD-L1 who showed a poor prognosis).

Regarding the background of T cells, at the time of diagnosis of DLBCL, only two cases (patients #1 and #4) showed a widespread and prominent CD3+/CD5+ population, while the other cases showed the sporadic infiltration of T cells (patients #3 and #4).

All patients achieved complete remission after first-line chemotherapy (10). One of them had a confirmed relapse of cHL and underwent ASCT, achieving complete remission of the disease before the development of DLBCL.

After the diagnosis of DLBCL, all 4 patients were treated with the R-CHOP regimen: one achieved complete remission and is still alive and disease-free, 147 months after the diagnosis of cHL, while the other 3 patients were not; 2 died within 6 months, while one achieved complete remission following the R-DHAP protocol followed by autologous and allogeneic stem cell transplantation. He is still alive and in complete remission after 74 months of follow-up from the diagnosis of cHL. Overall, the median overall survival (OS) from the time of cHL diagnosis was 6.3 years, while the median OS measured from the time of DLBCL diagnosis was 2 years.

## 4. Discussion

cHL is the paradigm of a curable disease; in recent decades, due to improvements in diagnostic techniques and advances in molecular biology and in the development of therapeutic options, the survival of these patients has significantly increased [11,12]. This has led to the need to monitor the occurrence of late complications, such as the appearance of second tumors, that dramatically affects the morbidity and mortality of these patients [6,7,8,9,13].

We performed a retrospective analysis of 269 patients with cHL diagnosed between 2007 and 2020; in 4 patients, DLBCL was diagnosed after a median of 53 months.

Although cHL, Hodgkin and Reed/Sternberg cancer cells (HRS) have only a few B-cell line markers, immunoglobulin gene rearrangement studies have shown that in more than 98% of cases, these cells are derived from mature B-cells at the germline phase of differentiation and contain the clonal rearrangement of the immunoglobulin heavy-chain variable region (IGHV) gene and the somatic mutation of the V region genes [3]. In addition, in some cases of cHL, HRS cells lack Ig gene transcription ability due to functional defects in the Ig gene regulatory elements. It has therefore been proposed that HRS cells originate from pre-apoptotic GCB cells with an acquired mutation that in normal conditions would have led to apoptosis [4].

Unlike cHL, in DLBCL the most common component is neoplastic, showing a diffuse proliferation of medium to large lymphoid cells with a distinct B-cell phenotype. Advances in trascriptome analysis have made it possible to identify two main DLBCL subgroups based on similarities with GCB cells (GCB-DLBCL) or with in vitro activated B cells (ABC-DLBCL) [1].

According to Hans’s algorithm, the COO of all 4 cases was non-GCB, in fact the neoplastic cells did not express CD10, while BCL6 was expressed in 3 cases. MUM1 was expressed in all cases. Instead, based on the gene expression profile, we found that 2 patients had GCB disease and 2 unclassified diseases, confirming the relevant discrepancy between the COO methods of subtyping. Notably, both patients with DLBCL identified on the NanoString as indeterminate died rapidly, while those classified as GCB survived.

In sequentially affected cHL and DLBCL patients, the crucial question is whether these lymphoid neoplasms are independent or correlated events. Applying immunoglobulin gene rearrangement analysis in cHL and DLBCL, Bellan et al. found identical rearrangements of DH and JH (during B cell ontogenesis this is the first segment of the immunoglobulin gene rearranged) and different rearrangements of VH [14]. In our study, IgH gene rearrangement analysis was not available, but, as shown by the Bellan et al. study, a common origin for large B cell and HRS cells in the 2 GCB subtype cases recognized by GEP analysis is conceivable. Instead, in our study, Hans’s algorithm seemed to rule out a common origin of the lymphoid neoplasia in these patients because all DLBCLs exhibited a non-GCB immunophenotype. Although Hans’s algorithm may in some cases be considered an acceptable alternative, it seems to be less accurate than molecular techniques for establishing the COO.

In situ hybridization of EBER was performed on the DLBCL samples to verify other similarities between DLBCL and cHL, considering that 30% of cHL are EBV positive. However, as also found in a previous study, none of our cases were positive to EBER and this finding would seem to exclude a possible pathogenic link between EBV and metachronous cHL/DLBCL [15].

Indeterminate potential clonal hematopoiesis (CHIP) shows that the acquisition of one or more somatic mutations in hematopoietic stem cells (HSC) leads to myeloid or lymphoid neoplasms. In several studies a role of CHIP has emerged in the recurrence and co-occurrence of the 2 different malignant lymphoid histologies in the same patient. However, unlike indolent lymphoid neoplasia, there is less evidence to support a stem cell origin of DLBCL [16]. Amini et al. reported a 12% prevalence of CHIP at the time of diagnosis of aggressive lymphomas, *DNMT3A* and *TET2* being the most mutated genes. They also reported an unfavorable outcome of patients with high-grade B-cell lymphoma (HGBCL) and CHIP in terms of both OS and progression-free survival (PFS) [17]. This association could suggest that CHIP promotes the survival of tumor cells, as well as possibly fostering a metachronous co-occurrence of cHL and HGBCL in the same patient. Indeed, the occurrence of mutations in driver genes (e.g., *DNMT3A* and *TET2*) in hematopoietic stem cells has also been reported in patients with cHL [18].

Beyond the intrinsic causes of the cHL-DLBCL co-occurrence, extrinsic causes could explain this event; all are linked to the post-treatment patient’s impaired immune system or to direct DNA damage to healthy cells.

As regards these factors, there are discordant results in the literature. In a large retrospective study, 26,826 patients with cHL were analyzed; among them, 259 had a diagnosis of secondary non-Hodgkin lymphoma (sNHL). In this study, it emerged that age, gender and disease stage at the time of the first diagnosis affect the likelihood to have sNHL [18].

A previous retrospective study analyzing the clinical-pathological features of composite and sequential lymphoma between CHL and DLBCL showed that composite cases had good outcome after an initial regimen while sequential cases did very poorly [19,20].

Swerdlow et al. found differences in the sNHL incidence between patients who had and had not undergone radiotherapy [7]. In our study, only one patient had undergone radiotherapy; this patient suffered a relapse of cHL treated with second-line therapy and ASCT. In a recent study by Eichenauer et al., age and LDH level at the time of diagnosis of cHL were independent risk factors for the development of sNHL, whereas gender and stage at diagnosis of cHL were not related to the sNHL risk [8].

In our case study the cumulative incidence of sNHL was 1.5%, in part comparable with the incidences found in the other studies. From the analysis of the clinical characteristics of our series, it is not possible to draw statistically significant conclusions due to the small number and the low incidence of onset; DLBCL occurred in three males and one female. Conversely neither older age nor advanced stage at cHL diagnosis were correlated with DLBCL incidence. In fact, median age at cHL diagnosis was 28 years; The three males were young (less than 40 years old) while the woman was 78 years old. The stage of cHL was 2 in 3 cases and 4 in 1 case. However, the exiguity of our cases does not allow for drawing any conclusion.

A higher risk of sNHL associated with splenectomy was found in a study of 3905 people with HL, treated between 1965 and 2000, who had survived for at least 5 years after treatment. This finding supports the hypothesis that immunosuppression could play an important role in this event. In the same study, it was also found that a high cumulative dose of procarbazine and irradiation of the mantle field were linked to a high risk of sNHL [9].

In our case series, three patients had a low chemotherapy load, having been treated only with ABVD, while one had undergone two lines of chemotherapy, radiotherapy and high-dose therapy followed by ASCT. No patient had undergone splenectomy.

The characteristics of the TME also have an important role in both cHL and DLBCL. It has been shown that in both cases the presence of tumor-associated macrophages (TAMs) and myeloid derived suppressor cells (MDSCs) is associated with a worse outcome and a greater likelihood of recurrence [21,22]. It was also observed that most of the PD-L1 expression in the TME of cHL was provided by the TAMs, facilitating HRS cells evasion of antitumor immunity [23].

Conflicting data are reported in the literature on the prognostic role of PD-L1 expression in DLBCL. A recent study by McCord and colleagues showed that PD-L1 does not identify high-risk patients, but it may be associated with a better prognosis in some patients with de novo DLBCL [24]. In other studies, a high expression of PD-L1 and soluble PD-L1 in de novo DLBCL was correlated with either a better or worse prognosis [25]. In our experience, PD-L1 was expressed in all cases at the time of diagnosis of cHL and in 2 of 4 cases in the DLBCL samples. In our series, both DLBCL patients with PD-L1 expression died. The discrepancies reported in literature could be attributable to the different patient populations studied or different PD-L1 reagents used.

Regarding the role of T cells in the pathogenesis of DLBCL, several studies have described that a lower percentage of CD3+ cells are related to bulky disease, elevated IPI, and advanced disease at diagnosis [26]. In our study, only two cases showed a widespread and prominent CD3+/CD5+ population, but it was not associated with a better outcome.

Therefore, human leukocyte antigen (HLA) typing and, possibly, the study of other baseline genetic characteristics in these patients could be useful to establish whether they can be considered predictive of the development and progression of the neoplastic process.

Furthermore, the interval between the diagnosis of cHL and of DLBCL, nanoString GEP and PD-L1 expression (the latter may be a possible therapeutic target) also seemed to influence the clinical outcome of patients. Large studies are necessary to verify the distinct roles of environmental factors, individual predispositions and constitutional or immune dysfunction consequent to previous treatment.

Due to the low incidence (only 4 patients out of 269—1.5%), it is not possible to draw statistically significant conclusions regarding the factors determining the occurrence of a DLBCL in patients with cHL. Furthermore, as highlighted, there are numerous factors that can manifest themselves at the beginning of this second diagnosis of lymphoma that in addition to the low incidence make it difficult to understand this anomaly.

## 5. Conclusions

In conclusion, the sequential cases from cHL to DLBCL performed very poorly, underlying the necessity to perform a second biopsy in primary refractory or disease relapsing cHL cases, to identify these patients. The origin of the second neoplastic cell in DLBCL patients with a previous history of cHL remains controversial, although the different immunophenotypic characteristics suggest that it may arise mainly de novo in a subject with a possible individual predisposition to lymphoid neoplasms, Clinically, it is important to maintain vigilance with patients with cHL even if it is in complete remission due to the possible development of sNHL.

## Figures and Tables

**Table 1:** Clinical, treatment, cell of origin, immunohistochemistry, and survival characteristics.

N.	Age	Sex	cHL Stage	Therapy for cHL	Months from cHL to DLBCL	DLBCL Stage	Therapy for DLBCL	DLBCLIPI	Survival from DLBCL (Months)	PD-L1 ex. in DLBCL	Cell of Origin (NanoString)	Hans Algorithm	Exitus
1	33	M	2B	ABVD	105	1B, bulky	R-CHOP	0	42	negative	GCB	non-GCB	no
2	18	M	2B	ABVD	24	1A	R-CHOP, R-DHAP ASCT, Allo-SCT	0	52	negative	GCB	non-GCB	no
3	23	M	2A	ABVD, RT, IGEV, ASCT	31	1B, bulky	R-CHOP	1	3	positive	Unclassified	non-GCB	yes
4	76	F	4B	ABVD	33	4B	Mini R-CHOP	3	6	positive	Unclassified	non-GCB	yes

RT: radiotherapy; ASCT: autologous stem cell transplantation; allo-SCT: allogeneic stem cell transplantation; M: male; F: female; cHL: classical Hodgkin’s lymphoma; DLBCL: diffuse large b cell lymphoma; GCB: Germinal Center B-Cell like.

## Data Availability

The datasets generated during the current study are available from the corresponding author on reasonable request.
